# Heart Rate Kinetics Response of Pre-Pubertal Children during the Yo-Yo Intermittent Endurance Test—Level 1

**DOI:** 10.3390/sports7030065

**Published:** 2019-03-15

**Authors:** João Brito, Luís Fernandes, António Rebelo, Peter Krustrup, Gustavo Silva, José Oliveira

**Affiliations:** 1 Portugal Football School, Portuguese Football Federation, Oeiras 1495-433, Portugal; 2 Center of Research, Education, Innovation and Intervention in Sport, Faculty of Sport, University of Porto, Porto 4200-450, Portugal; luisantoniocfernandes@gmail.com (L.F.); anatal@fade.up.pt (A.R.); 3 Department of Sports Science and Clinical Biomechanics, SDU Sport and Health Sciences Cluster (SHSC), University of Southern Denmark, Odense 5230, Denmark; pkrustrup@health.sdu.dk; 4 Research Center in Sports Sciences, Health Sciences and Human Development, CIDESD, University Institute of Maia, ISMAI, Maia 4475-690, Portugal; gugonsilva@gmail.com; 5 Research Centre in Physical Activity, Health and Leisure (CIAFEL), Faculty of Sport, University of Porto, Porto 4200-450, Portugal; joliveira@fade.up

**Keywords:** youth, health, endurance, cardiorespiratory fitness, Yo-Yo IE1

## Abstract

This study analyzed heart rate (HR) kinetics during the Yo-Yo Intermittent Endurance Test—level 1 (Yo-Yo IE1) in children. At the middle of the school year, 107 boys (7–10 years old) performed the Yo-Yo IE1. Individual HR curves during the Yo-Yo IE1 were analyzed to detect an inflection point between an initial phase of fast rise in HR, and a second phase in which the rise of HR is slower. The 7^th^ shuttle of the test was established as the inflection point. Engagement with extra-school sports practice was identified. Percentile groups (P_1_, P_2_ and P_3_) were created for body weight and physical fitness data composite (PF_composite_). Differences were found between the slopes of P_1_ and P_3_ on phase 1 for body weight (12.5 ± 2.7 vs. 13.7 ± 2.0 bpm/shuttle; *p* = 0.033; *d* = 0.50) and PF_composite_ (14.2 ± 2.5 vs. 12.5 ± 2.0 bpm/shuttle; *p* = 0.015; *d* = 0.75). Time spent >95% of peak HR was longer for the children engaged with extra-school sports practice (335 ± 158 vs. 234 ± 124 s; *p* < 0.001; *d* = 0.71); differences were also detected for PF_composite_ (P_1_, P_2_ and P_3_: 172 ± 92, 270 ± 109, and 360 ± 157 s, respectively; *p* < 0.05; *d* = 0.66–1.46). This study indicates that physical fitness and body weight influence HR kinetics during the Yo-Yo IE1 in pre-pubertal boys.

## 1. Introduction

The Yo-Yo tests [1] were introduced as a tool to monitor the capacity to perform and to recover from intermittent exercise, as opposed to the established laboratory and field tests that were the common standard procedure until the beginning of the decade of 1990, measuring mainly continuous exercise [2,3]. The Yo-Yo tests consist of repeated running shuttles performed at progressively increasing speeds, combined with short active rest periods, and performed until exhaustion [4]. Since their introduction, the tests have been widely used to evaluate intermittent exercise in sports given its capability to measure fitness performance [4,5,6].

Concurrently, the Yo-Yo tests are valid and reliable means of testing cardiovascular fitness in children under the age of 10 years old [7,8,9]. The actual increase of sedentary behaviors, which contributes to increased cardiovascular risk profiles [10,11], along with the pronounced increase on childhood obesity and decrease in physical fitness in children [12,13], made regular assessment of cardiorespiratory fitness as a key indicator to monitor the development of physical fitness in children. Also, regular cardiorespiratory fitness testing provides hints on the appropriate exercise stimuli that prevents the risk of developing overweight and/or obesity and cardiovascular disease in adulthood [8]. The Yo-Yo tests are inexpensive, simple to use, and allow the testing of up to 15 individuals simultaneously [1]. Therefore, their application on school-aged children to monitor cardiorespiratory fitness might be of interest, and a deep understanding of these tests is of major relevance. Therefore, studies have been conducted on the tests’ reliability, validity, and the relative influence of age, gender, and physical fitness in the Yo-Yo tests [7,8,14,15].

The kinetics of exercise testing has also been subject of study, with a particular emphasis on the analysis of oxygen uptake [16,17]. However, few studies have focused on the kinetics of heart rate (HR) during exercise testing in children. HR measurements are easier to apply than ventilatory testing. The equipment for HR measurements is cheaper and less complex to use during exercise and have minimal influence on performance and comfort of individuals. Moreover, HR is an effective means of evaluating cardiorespiratory fitness during sub-maximal exercise [8]. Therefore, a better understanding of HR kinetics of children during exercise testing is of utmost interest. Thus, the current investigation aimed to test intermittent endurance in pre-pubertal boys using the Yo-Yo Intermittent Endurance Test—level 1 (Yo-Yo IE1) and analyze the kinetics response of HR throughout the test.

## 2. Materials and Methods

### 2.1. Participants

The participants were recruited in six elementary primary schools (third and fourth grade) in the municipality of Braga, Portugal [n = 107 boys; age, 8.5 ± 0.8 (range: 7.8–9.3) years; height, 132.3 ± 8.1 cm; weight 34.2 ± 8.7 kg; and body mass index 19.3 ± 3.4 kg/m^2^]. The study design and procedures were in accordance with ethical standards and the Declaration of Helsinki. The study was approved by the Ethics Committee from the Faculty of Sports, University of Porto (CEFADE 18.2017). Also, the study has been approved by the director of each school. The children were informed about the aims of the study; parents or legal guardians provided written informed consent.

### 2.2. Testing Procedures

The measurements took place at the middle of the school year (February 2011). All children were engaged with complementary physical education (PE) classes in school, two times per week. Normally, school-based PE classes lasted for 45 min, and consisted of a general warm-up, followed by coordination/skill drills, small-sided team-sport games (e.g., soccer, basketball, handball), gymnastics, or general recreational physical activities. The emphasis of the PE classes was devoted to several different sports, according to the National program for PE. Additionally, information about extra-school physical activity engagement during the five months prior to the start of the study was collected via individual interviews with each child. Given that soccer was the most popular sport in the region [18], the sample was divided in two groups: children that had been engaged with regular and oriented after-school soccer practice in the five months prior to the study, with two weekly 60-min practice sessions (n = 37), and children not engaged with any extra-school sports activity (n = 70). Overall, the extra-school soccer training sessions consisted of a general warm-up, followed by technical exercises and several small-sided soccer games.

The data were collected in two sessions, with an interval of two days apart. The first session was devoted for assessing anthropometry, sprint and jump testing; a second session focused on cardiorespiratory fitness testing. During the anthropometric assessments, children wore light clothing and no shoes. Height was assessed with a stadiometer (model 708, Seca, Hamburg, Germany). Weight and percentage of body fat were evaluated with a bioimpedance scale (Tanita Inner Scan BC-532, Tanita, Hoofddorp, The Netherlands). The average of duplicate measures was considered for analysis. Fitness tests were conducted on an indoor multi-sports ground. The children performed a 12-min warm-up routine consisting of light jogging and stretching, and familiarization trials of the test prior to any fitness testing. Speed was assessed with a 15-m sprint test with three pairs of photoelectric cells (Speed Trap II, Brower Timing Systems, Draper, UT, USA) positioned at the starting line, and at 5 m (5-m sprint) and 15 m (15-m sprint) from the starting line. Children were instructed to run as fast as possible from a standing position 30 cm behind the starting line. The better (fastest) of two trials was retained for analysis. Jumping height was assessed with a countermovement jump test (CMJ) on a jumping mat (Digitime 1000, Digitest, Jyvaskyla, Finland). Children were instructed to stand erect; then, after flexing the knees to a squat position, they jumped vertically as high as possible maintaining hands on hips. Two trials were given; the better of two CMJ was retained for analysis. The Yo-Yo IE1 was used to evaluate cardiorespiratory fitness [1], and to assess submaximal and maximal HR values. An adapted version of the Yo-Yo IE1 has been validated to test cardiorespiratory fitness in children of this age [7]. During the test, the participants wore HR monitors (Polar Team System^TM^, Polar Electro OY, Kempele, Finland). The test consisted of repeated 2 × 20-m shuttle runs at progressively increased speeds and 5-s period of rest between shuttle runs. The test was controlled by audio bleeps from a MP3 player. The aim was to perform as many shuttle runs as possible; when the child failed twice to reach the finish line on time, the distance covered was recorded and used as the test result. Only one trial was given.

### 2.3. Statistical Procedures

**Figure 1:**
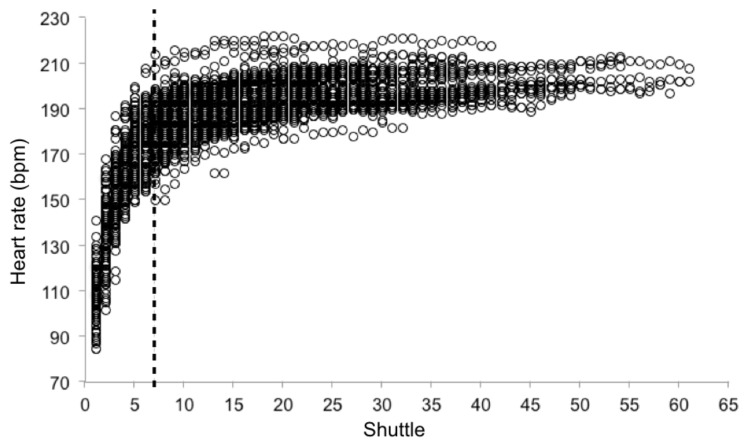
Absolute heart rate (bpm) during the Yo-Yo IE1 test (n = 107 boys). Vertical dotted line represents the seventh shuttle run.

Descriptive statistics (mean ± standard deviation) were calculated. A composite score (PF_composite_), based on Z scores calculated for all physical fitness tests, was determined to provide a more complex indicator of physical fitness. To compute PF_composite,_ 5- and 15-m sprint performances were reversed, because lower times reflect better performance. Individual peak HR (HR_peak_) during the YoYo-IE1 was determined to establish absolute and relative time spent above 95% of HR_peak_ during the test. Individual HR curves of the Yo-Yo IE1 tests were analyzed to detect the inflection point between an initial phase of fast rise in HR values (phase 1), and a second phase in which the rise of the HR values is much smoother (phase 2). The inflection point was detected through a visual analysis of dispersion plots containing information from the entire sample (Figure 1). The seventh shuttle run was established as the inflection point between the two phases of the test. The slope (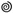) values for the two phases was obtain with linear regression models.

Differences between children engaged with soccer and children not engaged with any extra-school sports activity were calculated using an independent-samples t-test. For comparisons, the group was also divided in three percentile groups: P_1_ (<percentile 33), P_2_ (percentile 33–66) and P_3_ (>percentile 66) for body weight (P_1_, n = 35; P_2_, n = 36; P_3_, n = 36) and PF_composite_ (P_1_, n = 36; P_2_, n = 35; P_3_, n = 35). Differences between the HR slopes of the three percentile groups were calculated using one-way ANOVA, with Bonferroni post-hoc comparisons to discriminate those differences. Statistical comparisons were conducted using IBM SPSS Statistics for Mac, version 20 (IBM Corp., Armonk, NY, USA). Significance was set to *p* < 0.05. Standardized differences in means (effect size, *d*) were calculated for comparisons. Effect sizes were established as trivial (*d* < 0.2), small (0.2 < *d* < 0.6) moderate (0.6 < *d* < 1.2), large (1.2 < *d* < 2.0), very large (2.0 < *d* < 4.0), nearly perfect (*d* > 4.0), and perfect (*d* = infinite) [19]. Pearson correlations (*r*) were calculated, and classified as trivial (*r* < 0.1), small (0.1 < *r* < 0.3) moderate (0.3 < *r* < 0.5), large (0.5 < *r* < 0.7), very large (0.7 < *r* < 0.9), nearly perfect (0.9 < *r* < 1), and perfect (*r* = 1) [19].

## 3. Results

Overall, 5m and 15m sprint performances were 1.34 ± 0.10 s and 3.26 ± 0.24 s, respectively; CMJ was 21.4 ± 4.3 cm. Absolute time and percentage of time spent above 95% HR_peak_ during the Yo-Yo IE1 for the total sample was 268 ± 144 s and 52.0 ± 14.0%, respectively. Descriptive values for body weight and PF_composite_ percentile groups (P_1_, P_2_ and P_3_) were 26.2 ± 1.9, 32.3 ± 2.0, 44.2 ± 7.0 kg, and −0.9 ± 0.4, 0.0 ± 0.2, 1.0 ± 0.4 arbitrary units, respectively. No correlation was observed between body weight and PF_composite_ (*p* = 0.181).

Moderate effect sizes were observed for absolute time spent above 95% HR_peak_ during the Yo-Yo IE1 between children engaged with extra-school soccer practice and children not engaged with any extra-school sports activity (335 ± 158 vs. 234 ± 124 s, *p* < 0.001, *d* = 0.71). Also, moderate to large effect sizes were detected between PF_composite_ percentile groups (P_1_, P_2_, and P_3_: 172 ± 92, 270 ± 109, and 360 ± 157 s, respectively; *p* < 0.05; *d*_1–2_ = 0.98; *d*_2–3_ = 0.66; *d*_1–3_ = 1.46) for absolute time spent above 95% HR_peak_ during the Yo-Yo IE1. However, no differences were found on absolute time spent above 95% HR_peak_ during the Yo-Yo IE1 for body weight. Likewise, no differences were found in relative time spent above 95% HR_peak_ during the Yo-Yo IE1 between children engaged with extra-school soccer practice and children not engaged with any extra-school sports activity, PF_composite_ percentile groups, and body weight percentile groups.

Overall, significant differences were detected in slope values of HR before vs. after the seventh shuttle run of the Yo-Yo IE1 (phase 1 vs. phase 2: 13.37 ± 2.50 vs. 0.96 ± 0.74 bpm/shuttle; *p* < 0.001; *d* = 6.73) for the entire sample. However, no correlations were detected between body weight and the slopes of HR data of phase 1 and 2 (*p* = 0.106 and *p* = 0.198, respectively). No significant correlation was found between the slope of HR data in phase 1 and PF_composite_ (*r* = −0.25; *p* = 0.09), but a moderate inverse correlation was detected between the slope of HR data in phase 2 and PF_composite_ (*r* = −0.32; *p* = 0.01).

**Figure 2:**
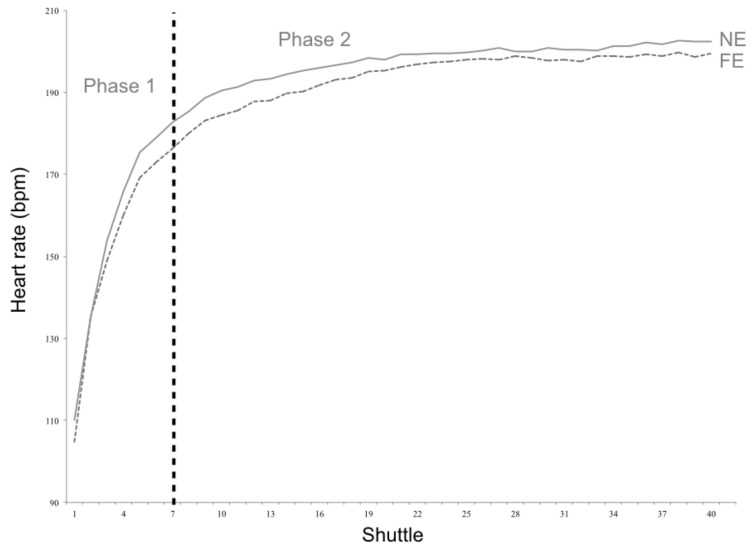
Heart rate curves for the children engaged with extra-school soccer practice (FE) and children not engaged with any extra-school sports activity (NE).

Figure 2 presents HR curves for the children engaged with soccer and children not engaged with any extra-school sports activity. No differences were found between the slopes of the two groups for either phase 1 (13.6 ± 2.7 vs. 13.0 ± 2.1 bpm/shuttle; *p* = 0.29) and phase 2 (1.0 ± 0.7 vs. 0.9 ± 0.8 bpm/shuttle; *p* = 0.30).

**Figure 3:**
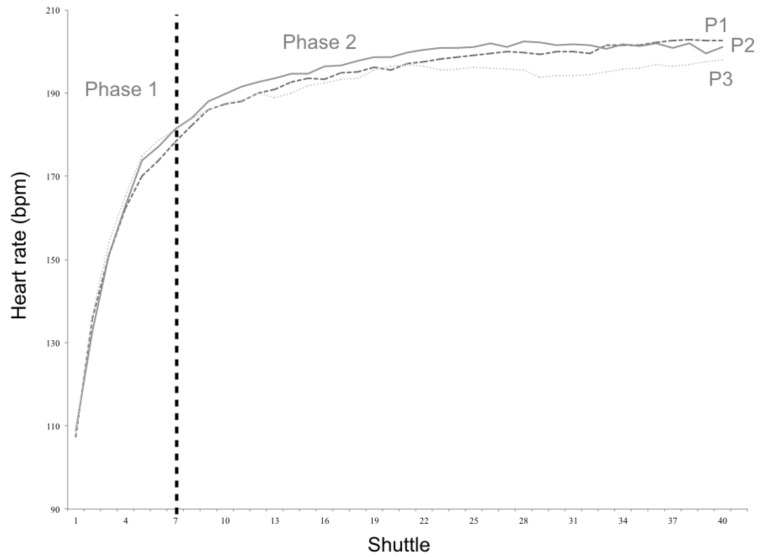
Heart rate curves for the three weight percentile groups (P_1_, P_2_, and P_3_).

**Figure 4:**
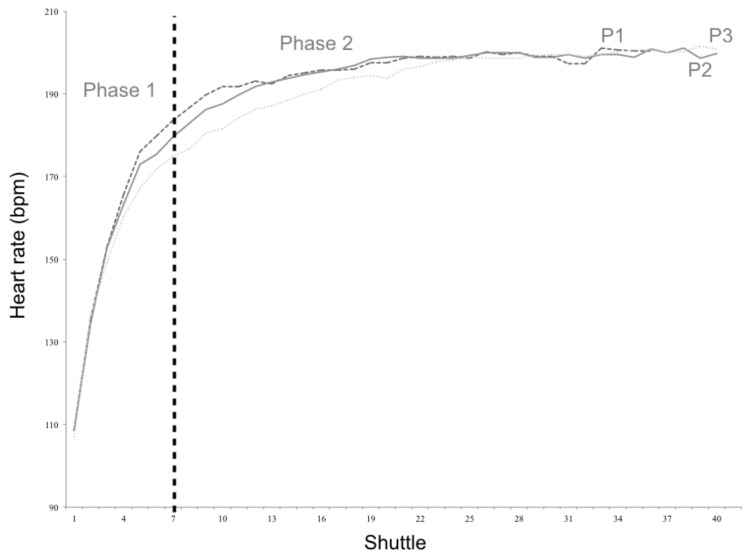
Heart rate curves for the three physical fitness percentile groups (P_1_, P_2_, and P_3_).

Figure 3 and Figure 4 present HR curves of the three percentile groups established for body weight and PF_composite_. Small effect sizes were detected between the slopes of P_1_ and P_3_ of body weight during phase 1 (12.5 ± 2.7 vs. 13.7 ± 2.0 bpm/shuttle; *p* = 0.033; *d* = 0.50). Also, moderate effect sizes were observed for the slopes of P_1_ and P_3_ of PF_composite_ during both phase 1 (14.2 ± 2.5 vs. 12.5 ± 2.0 bpm/shuttle; *p* = 0.015; *d* = 0.75) and phase 2 (1.2 ± 1.1 vs. 0.7 ± 0.3 bpm/shuttle; *p* = 0.021; *d* = 0.62).

## 4. Discussion

The present study aimed to analyze the kinetics of HR response to the Yo-Yo IE1 in children under the age of 10. A separation was detected between an initial phase of fast rise in HR values (phase 1), and a second phase of much smoother rise of the HR values (phase 2). The seventh shuttle run was recognized as the inflection point between the two phases of the test and significant differences were detected in slope values between the two phases.

There is a lack of studies on the analysis of HR kinetics during exercise testing in children. Interestingly, the kinetic profile of HR observed in the present study is in accordance with several studies focusing on oxygen uptake kinetics [16,17,20,21]. In fact, moderate to large correlations between HR and VO_2_ have been identified during field and laboratory activities [6,15,22], and strong relationships were also identified between the ventilatory threshold and the HR deflection point [23,24]. Altogether, this might explain the similarities found on both HR kinetic profiles.

Engagement with regular extra-school soccer practice did not prove to be a differentiating variable regarding both phase 1 and 2 slopes even though the individuals engaged with extra-school soccer practice presented slightly lower values of HR on both Yo-Yo IE1 phases. However, PF_composite_ percentile groups presented distinct slopes for phase 1 and phase 2, indicating that higher physical fitness might correspond to lower slopes for both phases of HR kinetics during the Yo-Yo IE1. Regular sports participation, namely soccer practice, as proven to induce significant improvements on physical fitness of young children [14,25,26]. Therefore, the lack of significant differences between children engaged with soccer and children not engaged with any extra-school sports activity might be explained by other factors, such as regular physical activity levels. In fact, the levels of physical activity throughout the year, namely the content of the PE classes and recreational sport activities, as well as maturational status were not effectively controlled, and stand as limitations of the current investigation.

The results also showed that body weight might influence HR kinetics during the Yo-Yo IE1. In fact, body weight percentile groups have presented distinct slopes for phase 1 of the test, showing that higher body weight is associated with a faster rise of the HR values on the first shuttles of the Yo-Yo IE1. Since lower body weight is usually associated with improved health and physical fitness [27], these results, together with the differences found between PF_composite_ groups, seem to indicate that fitter and healthier children have a slower rise in HR values during the Yo-Yo IE1. These assumptions are partially supported by small and moderate correlations found between PF_composite_ and the slope of phase 1. 

Absolute time and percentage of time spent above 95% HR_peak_ during the Yo-Yo IE1 were also subject to analysis. Distinct behaviors for both variables were detected. Body weight did not influence absolute time spent above 95% HR_peak_ during the Yo-Yo IE1. However, significant differences were found between children engaged with soccer and children not engaged with any extra-school sports activity and PF_composite_ percentile groups. In fact, individuals with higher physical fitness are expected to perform better during the test, meaning a higher absolute time spent to complete it and, consequently, more time spent above 95% HR_peak_.

As any maximal test, it is also expected that the later stages of the Yo-Yo IE1 be performed at high intensity [1,15]. Lactate threshold of well-trained individuals usually occurs at around 90% of HR_peak_ [28]. Thus, the higher absolute time spent above 95% of HR_peak_ might indicate a superior anaerobic capacity of the children engaged with regular soccer practice. Yet, the analysis of the percentage of time spent above 95% HR_peak_ during the Yo-Yo IE1 provides different results. No differences were found for percentage of time spent above 95% HR_peak_ during the test between children engaged with soccer and children not engaged with any extra-school sports activity, PF_composite_ percentile groups and body weight percentile groups. This might indicate that the test requires the same relative intensity to be performed, regardless of the individual’s engagement with regular physical activities, physical fitness, or body weight.

## 5. Conclusions

The Yo-Yo IE1 is a high intensity demanding activity, requiring high aerobic and anaerobic capacity, regardless the children’s engagement with regular physical activities, physical fitness or body weight. Submaximal intermittent testing might be appropriate in children. Overall, children with lower body weight might present a slower rise of the HR during submaximal exercise. Moreover, children with better physical fitness might present lower slopes of HR kinetics during both submaximal and maximal exercise. Altogether, the results of the present study indicate that PE teachers and practitioners dealing with exercise testing in children should be aware that physical fitness and body weight influence HR kinetics during the Yo-Yo IE1 in pre-pubertal boys.
